# A retrospective evaluation of 182 free flaps in extremity reconstruction and review of the literature

**DOI:** 10.3205/iprs000162

**Published:** 2022-01-14

**Authors:** Sergey Arakelyan, Emrah Aydogan, Nick Spindler, Stefan Langer, Olimpiu Bota

**Affiliations:** 1 Department of Orthopedics, Trauma and Plastic Surgery, University of Leipzig, Germany; 2 University Center of Orthopaedic, Trauma and Plastic Surgery University Hospital Carl Gustav Carus, TU Dresden, Germany

**Keywords:** free flap, reconstructive surgery, risk factor, microsurgery, microvascular anastomosis, extremity reconstruction

## Abstract

**Introduction**: During the past decades, free flaps have been given a central role in the reconstructive surgery. Especially in the extremities, where there is a scarcity of available tissues for local flaps, free flaps play a central part. The aim of this study was to evaluate the risk factors contributing to partial and total flap failure.

**Patients and methods**: In a retrospective cohort study, all data concerning patients who underwent free flap reconstruction of the extremities during the first five years since the founding of the department of plastic surgery were gathered. Patient- and surgery-related risk factors were analyzed in correlation to the postoperative complications.

**Results**: In total, 182 free flaps were included in this study. Partial and total flap failure were noted in 21.42% and 17.03%, respectively. A correlation was seen between the time lapsed from debridement until flap coverage, with flaps performed between day 4 and 14 having the least quote of flap failure (p=0.022). Gender, age, arterial hypertension, nicotine abuse, diabetes mellitus, peripheral arterial disease and the number of anastomosed veins were not significantly associated with free flap failure.

**Conclusion**: Our study showed that free flaps can be safely performed in healthy patients as well as in patients with risk factors, with an acceptable flap loss rate. Randomized controlled studies are needed to clarify the exact role of each risk factor in free flap surgery.

## Zusammenfassung

**Einleitung**: Während der letzten Dekaden erhielt die freie Lappenplastik eine Hauptrolle in der rekonstruktiven Chirurgie. Insbesondere an den Extremitäten, mit begrenzt verfügbarem Gewebe, haben freie Lappenplastiken eine besonders wichtige Funktion. Das Ziel unserer Studie war, die Risikofaktoren für einen partiellen oder kompletten Lappenverlust zu evaluieren.

**Patienten und Methoden**: In einer retrospektiven Kohortenstudie wurden die Daten von allen Patienten gesammelt, welche eine freie Lappenplastik innerhalb der ersten fünf Jahre seit der Gründung unserer Abteilung erhielten. Patienten- und Chirurgie-bezogene Risikofaktoren wurden bezüglich der postoperativen Komplikationen untersucht.

**Ergebnisse**: Insgesamt wurden 182 freie Lappenplastiken in diese Studie eingeschlossen. Ein partieller Lappenverlust trat bei 21,42%, ein kompletter bei 17,03% auf. Die Zeitspanne von Débridement bis zum Tag der Deckung mittels freier Lappenplastik stellte einen statistisch signifikanten Risikofaktor für den Lappenverlust dar (p=0.022). Die Lappenplastiken, die zwischen Tag 4 und 14 operiert wurden, zeigten die geringste Verlustquote. Geschlecht, Alter, arterielle Hypertonie, Nikotinabusus, Diabetes mellitus, periphere arterielle Verschlusskrankheit sowie die Anzahl der anastomosierten Venen waren nicht signifikant mit dem Lappenverlust assoziiert.

**Schlussfolgerung**: Unsere Studie zeigt, dass freie Lappenplastiken sicher und mit einer akzeptablen Lappenverlustratte sowohl bei Gesunden als auch bei Risikopatienten durchgeführt werden können. Randomisiert kontrollierte Studien sind zukünftig notwendig, um die genaue Rolle jedes Risikofaktors festzustellen.

## Introduction

Over the last decades, microvascular free flap surgery became the therapeutic gold standard to cover complex three-dimensional tissue defects [1], in the treatment of extremity osteomyelitis [2], to avoid imminent amputation cases [3] or for reconstruction after tumor resection [4]. The constant improvements of the microsurgical instruments and techniques have helped to reduce the revision and flap failure rates over the years. Nevertheless, as a free tissue transfer implies a lesion to the vessel intima, a small, but persistent risk of vessel thrombosis exists [5]. Several patient-related risk factors like diabetes, peripheral arterial disease, radiotherapy, or infection may increase the risk of partial or total flap failure. The timely recognition of these risk factors may reduce the complication and revision rates [6]. Except for the patient-related risk factors, several other factors related to treatment, like anesthesia time or operative technique may influence the outcome.

Since its first description by Jacobson and Suarez [7], the vessel couplers have been developed and introduced in the clinical practice for the venous anastomosis, as the soft, pliable venous wall enables the use of these devices [8]. Venous couplers have proven over time a reduced flap failure risk and especially a reduced operative time, meaning a reduced flap ischemia time and the possibility of swiftly performing more venous anastomosis [9]. The question remains though if more venous anastomosis could reduce the risk of partial or total flap failure [10].

The aim of our study was to investigate the risk factors for partial or total flap failure in a large patient cohort, operated in a university setting.

## Patients and methods

The conduct of our study was approved by the ethics committee at the medical faculty of the university.

We retrospectively investigated all free tissue transfers for extremity reconstruction performed in our institution in the first five years since the establishment of the department of plastic surgery. The data was collected from the medical files. Preoperative, intraoperative and postoperative possible risk factors were gathered in Excel 365 (Microsoft Corporation, WA, USA). The follow-up period was three to 12 months. 

The statistical evaluations were carried out using JASP version 0.15 (University of Amsterdam, The Netherlands). A value of p<0.05 was assumed to be statistically significant for all statistical tests.

## Results

We identified 182 free flaps in 167 patients. 13 patients received a second flap to complete the reconstruction. Another patient underwent three subsequent microvascular free flap reconstructions. The patient population consisted of 29% women and 71% men. There was no significant influence of the gender of the operated patient on the presence of flap failure (p=0.104). The average patient age was 56.1 years (10–89). On average, the men were about 10 years younger than the women on the day of the operation. The World Health Organization (WHO) classification was considered when dividing the patient collective into two groups with patients who have not yet reached the age of 60 and those aged 60 years or more. 41.2% of patients were 60 years of age or older, with no significant differences concerning partial or total flap failure (p=0.624) compared to patients below 60 years.

The duration of the operation was defined as the incision-suture time and averaged 283 (151–569) minutes, including the time for wound debridement and osteosynthesis procedures. No statistically significant correlation could be established between surgery time and postoperative complications (p=0.410).

**Figure 1:**
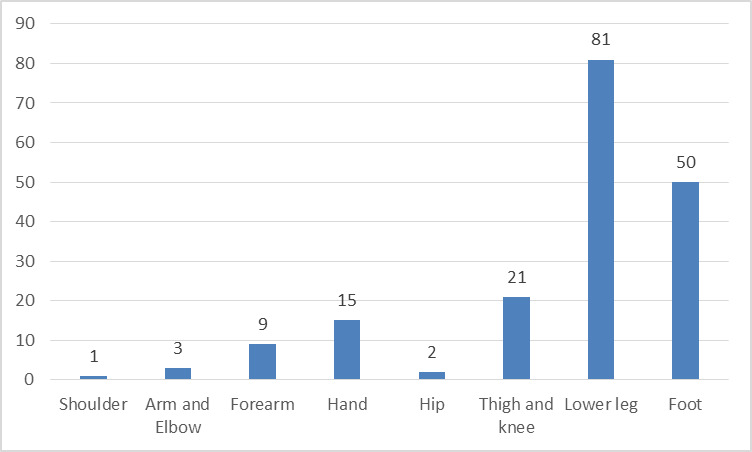
Patient distribution after reconstructed body region

The most frequently performed flap was the anterolateral thigh flap (ALT) with 98 flaps. The free latissimus dorsi flap (LDMF) was the second most transferred flap, 68 patients were receiving such a flap in our study. Six of these flaps were harvested as muscle sparing flaps. Further, there were six lateral arm flaps, three deep inferior perforator DIEP flaps and three patients were treated with vastus lateralis flaps. One rectus femoris, one rectus abdominis, one gracilis and one parascapular flap were also transferred as microvascular free flaps (Table 1 (Tab. 1)). In terms of the recipient region, 28 flaps were used to reconstruct the upper extremity and 154 flaps were used to reconstruct the lower extremity (Figure 1 (Fig. 1)). There was no significant correlation to total flap loss (p=0.501).

In terms of flap-related complications, there were partial flap necrosis in 39 patients and complete flap loss in 31 patients (Table 2 (Tab. 2)). Most of the partial flap necrosis could be treated conservatively. In seven patients a hematoma had to be operatively removed. In eleven cases a revision of the vascular anastomoses was performed.

**Figure 2:**
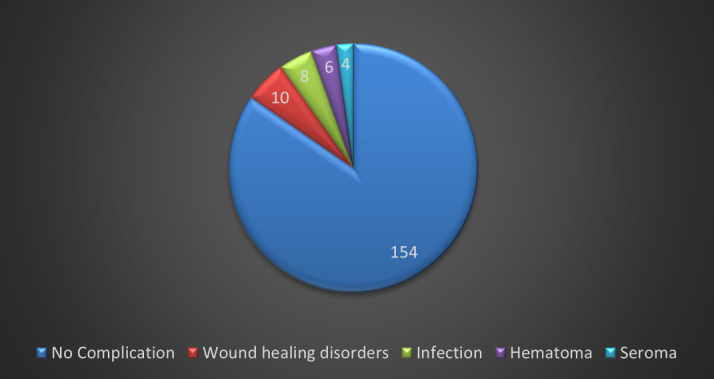
Analysis of donor site complications

Concerning the donor site, 154 cases (85%) showed now postoperative complications, whereas 28 cases (15%) developed a complication at the donor site, including infection, wound healing disorders, hematoma and seroma (Figure 2 (Fig. 2)).

**Figure 3:**
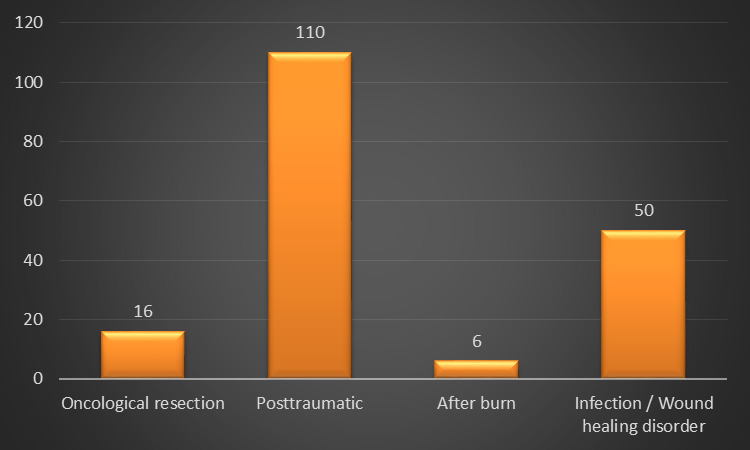
Etiology of soft tissue defect

The etiology of the soft tissue defect could be divided in posttraumatic, oncologic, infection and wound healing disorders and after burn trauma (Figure 3 (Fig. 3)). There was no statistical significance concerning total flap failure (p=0.946). The mean length of stay in hospital was 34.9 (4–228) days. 

The timing of the free flap operation was also considered, depending on the time elapsed from the injury to the free flap. After oncological resection, the time of the last resection is evaluated until the day of the surgical intervention. With this consideration, soft tissue defects were divided into three temporal categories. 39 patients received free flaps within the first 72 hours, 62 patients between the 4^th^ and 14^th^ day and 81 patients from the 15^th^ day onwards (Table 3 (Tab. 3)). On average, the free flap was transplanted after 19.01 days. A significant correlation was determined between the period of time elapsed until flap coverage and flap loss (p=0.022), with the best results being obtained in the group which was operated between day 4 and day 14.

Furthermore, the flaps were sorted according to the day of the week, on which the operation was performed. There was no statistically significant difference between the operation days concerning partial or total flap failure (p=0.134) (Table 4 (Tab. 4)). An analysis of the yearly distribution of flaps was also performed to investigate if the learn curve could be a contributor to the flap loss, but no statistical significance was found (p=0.911).

Twenty-one patients had a bone gap and needed a spacer and secondary bone reconstruction. Four of these patients developed a complete flap failure (p=0.794).

At the time of the free flap surgery, 73.6% of the patients had a Body Mass Index between 18.5 and 24.9, 11% had a BMI between 25 and 29.9, being designated as overweight and 15.4% patients had obesity with a BMI between 30 and 49.86. There was no significant association of BMI with postoperative flap failure (p=0.921).

Arterial hypertension was recorded in 30% of the patients as a secondary diagnosis. Arterial hypertension did not show a significant influence on the occurrence of flap failure (p=0.567) in the analysis of our patients.

Routinely alcohol abuse was documented in 60 patients. Seven of these patients developed a complete flap failure (P=0.177). A total of 29.67% of patients were heavy smokers, while the rest of the patients belonged to the non-smoking group. Nicotine abuse showed no significant influence on flap failure (p=0.605).

There was a high incidence of diabetes mellitus in the investigated cohort (39%). Nevertheless, there was no significant influence of this condition on flap failure (p=0.658). 26 patients had the established diagnosis of peripheral arterial disease. Five of these patients developed a complete flap necrosis (p=0.758). Eleven patients had documented deep vein thrombosis or varicose veins in their medical history. None of these showed a flap failure. 

The number of anastomoses were also investigated. Two artery anastomoses were performed in six flaps, as a flow-through flap. In 54 cases one venous anastomosis was performed, in 124 flaps two venous anastomoses were performed and in 4 cases three venous anastomoses were performed. In our patient group, there was no statistically significant correlation between the number of venous (p=0.218) or arterial anastomoses (p=0.265) and the occurrence of flap failure. 

In 10 patients vein grafts were employed to ensure the blood supply to the soft tissue defect. Out of these, six flaps showed a complete necrosis. The use of vein grafts appeared to be a highly significant risk for a complete flap failure (p<0.001).

The free flaps were also divided into an end-to-end and an end-to-side group according to the respective microsurgical technique of arterial anastomosis. In 40.6% of the cases, the arteries were microvascular anastomosed in end-to-end technique. In 59.4% of the free flaps, the end-to-side technique for arterial connection was selected. 23% of the end-to-end anastomoses eventually developed a total flap necrosis, compared to only 13% of the end-to-side anastomoses (p=0.078). Veins were mostly anastomosed with the coupler in an end-to-end technique.

## Discussion

In this study we retrospectively investigated 182 free flaps in the reconstruction of extremity soft tissue defects, performed in the first five years after the foundation of our plastic surgery department. The current pooled data in the literature shows an incidence of partial and complete flap loss of 8% and 6% for the upper extremity and 6% for both in the lower extremity [5], [11], although there are studies in multimorbid patients with a reported flap loss rate of up to 26.47% [12]. The partial and complete flap loss in our study were 21.42% and 17.03%, decidedly more than the literature average. The focus of the study was to determine which factors favor the development of a partial and especially complete flap loss.

Sanati-Mehrizy et al. [13] found after reviewing 1,921 free flaps for reconstruction of all body regions that BMI and male gender are independent risk factors for flap loss. With the growing age of populations worldwide, the indication for free flaps in elderly patients has also been constantly growing. In a meta-analysis, Üstün et al. [14] found no significant differences in flaps success rate and complication rates in elderly patients. In a study on 5,951 patients, Jubbal et al. [15] found age not to influence the surgical or medical complications, the flap failure or the reoperation rates. They found though BMI and prolonged operative time to have a significant impact on surgical complications. In our study, we found no significant impact of gender, age or BMI on the flap survival rate. The operating time hasn’t shown either a significant impact on flap survival. The reported impact of prolonged operating times on the outcome may reflect an assiduous flap harvest and prolonged flap ischemia times. In extremity surgery the operating time reflects not only the flap surgery time, but also the elapsed time for bone reconstruction. This could explain why in our study the flap survival did not appear to be directly influenced by the total operative time.

The vessel status is known to have an important impact on flap survival. As free flaps imply a lesion of the vessel intima, a small risk of postoperative thrombosis persists despite the progress that microsurgery made over the years. If the recipient vessels show a preexisting condition, the risk of postoperative thrombosis may increase even further. Peripheral arterial disease is one of the most influential factors concerning free flap surgery. The lack of permeable recipient vessels is usually a contraindication for free flap surgery. In cases where a diseased, though permeable vessel can be isolated, a free flap can be transferred, though with increased complication and flap loss rates [12]. The incidence of peripheral arterial disease appears to increase after the age of 65 [16]. Venous affections may also have an impact on flap survival. Deep venous thrombosis and varicosis veins may complicate the free flap transfer and a preoperative accurate diagnosis should be performed in patients at risk [17], [18]. Although expected, we couldn’t find a correlation between arterial or venous disease and flap failure in our study. One reason could be the underdiagnosis of atherosclerosis, as a preoperative distraction angiography of the reconstructed extremities was not the standard of care.

Smoking has traditionally been incriminated for failure of microsurgical interventions, attributed to the vascular spasm and to delayed wound healing. The evidence hasn’t proven though that nicotine abuse would be a risk factor for free flap failure [19]. The general recommendations remain nevertheless that patients should strictly abstain from nicotine about six weeks before and four weeks after the surgical procedure [20]. Regardless, smoking should not be a contraindication for free flap surgery, especially in non-elective surgery.

In the study cohort there was an increased percent of patients with diabetes mellitus. The statistical analysis hasn’t shown a correlation with flap failure. This result is consistent with the literature. In a study on 6,030 patients, Kantar et al. found diabetes mellitus to increase the risk of wound healing disorders, wound dehiscence and prolonged hospital stay, but found no correlation to flap failure [21]. Furthermore, free flaps can be used successfully for salvage of diabetic feet, with the end-to-side anastomosis being recommended to avoid worsening the foot ischemia and wound healing disorders [22]. Another study on diabetic feet also showed an increased flap loss rate of 21.7%. Nevertheless, microsurgical reconstruction in patients with extensive tissue defects due to diabetic ulcers represents the possibility of restoring independent mobility. In patients with successful reconstruction, the mean limb recovery rate and independent mobility rate were beneficial [23].

The free flap armamentarium that a reconstructive surgeon can use is overwhelming. Nonetheless, based on availability, ease of harvest and donor site morbidity, the ALT has evolved to become the workhorse of reconstructive surgery, not only in the extremities, but also in the head and neck and other body regions [24], [25]. A limitation of the ALT flap would be in overweight patients, where the thick subcutaneous tissue makes the reconstruction of thin, soft tissue defects in the lower leg, foot, or hand difficult. The primary thinning of the flap can ease the shaping and the sewing in of the flap with the price of partial flap devascularization and necrosis [26]. A systematic review from Sharabi et al. showed that flap thinning may be safer in smaller flaps, whereas the thinning of large ALT flaps may be associated with higher necrosis rates [27]. Although with less aesthetic results and more donor-site morbidity, the LDMF remains a reliable flap choice, especially in patients with extended soft tissue defects [28] or as mentioned, when the ALT is not a feasible option. The muscle sparing LDMF was proved to reduce the donor site morbidity, including seroma formation [29]. In our study, we proved that ALT and LDMF remain the two most feasible flaps for extremity reconstruction, with the muscle sparing LDMF as an alternative in appropriate cases. 

With regard to the correct timing of free flap surgery, Godina recommended in 1986 that tissue defects should be covered with a free flap within 72 hours [30]. He made this decision after conducting his own investigations on 532 patients and their free flaps on the lower extremities. Currently, a meta-analysis of 43 previous studies analyzed the complication rate in relation to the time from tissue injury to surgery. Early surgical intervention within the first three days should significantly reduce the rate of flap failure and infection [31]. Patients undergoing microsurgery in the early period had a significantly lower rate of infection, a shorter length of stay in hospital and an overall lower number of surgical interventions. However, there were no differences in the indication for amputation or flap failure [32]. Differently, a recent study has shown that flaps can be safely performed up to 10 days after injury without increased risk of flap failure [33]. In another study, monitoring of 88 free flaps over a period of eight years did also not show significantly higher flap complication rates either if microsurgical reconstruction with free flap was performed after the first 72-hour time interval. Rather, the effective adaptation of the pre- and perioperative treatment to the individual patient would be crucial for a successful outcome [34]. A systematic review of 463 free flaps used for microsurgical reconstructions of the upper extremities showed no strict evidence for a significantly lower complication rate of free flaps, when comparing different time intervals subdivided into within 24 hours, within five days, between the sixth and 21^st^ day and after the 21^st^ day. However, earlier reconstruction can shorten the length of hospital stay and limit the associated medical costs [35]. Our study showed in a statistically significant manner that a free flap coverage performed between days 4–14 from the initial debridement or resection had better results concerning complete flap loss.

Thus, effective surgery of a free flap reconstruction is safely possible even after 72 hours. It is also recommended that the microsurgical procedure be preceded by stabilization of the injury, clean debridement and optimal dressing care. In times of negative pressure wound therapy (NPWT) careful planning of a vascular anastomosis is preferable to the fastest possible tissue coverage [36]. A meta-analysis compared 928 cases of NPWT with 930 treatments without NPWT. NPWT significantly reduced the rates of dehiscence, hematoma, seroma, skin necrosis and bleeding [37]. NWPT thus plays an important role in the treatment of tissue defects. It ensures initial wound sealing, allowing debridement, vascular diagnostics and extremity perfusion to be optimized until the free flap is performed.

Some studies showed higher complication rates (17.1% vs. 6.2%) in the lower extremities than in the upper extremities [38]. This finding could not be confirmed in our study. The key message here is that intensive flap monitoring in a special microsurgical intensive care unit by qualified nurses and experienced surgeons allows early detection of vascular compromise, which leads to better results [39]. Bigdeli et al. showed in 581 flaps a re-exploration rate of 14.8%, with a salvage rate of 75.6% [40]. The authors showed that the mean time from tissue transfer to re-exploration was 16.2 ± 1.9 hours and the time from diagnosis of perfusion insufficiency until re-exploration was 1.3 ± 0.4 hours. Our study describes the performed free flaps during the first five years from the establishment of the department. Although the statistical analysis didn’t show an improvement in flap loss rate with the learning curve, an efficient flap monitoring system and a swift transfer to the operation room need to be established. Our analysis of the flap failure rate according to the day of the week didn’t show any correlation.

Another debate in the literature focuses on the anastomosis of one or more veins. Some argue against a second venous anastomosis with the well-known argument about a reduced venous flow rate and thus a higher thrombosis and flap failure rate [41]. Taking a closer look at hemodynamics, it can be seen that the venous flow velocity is in fact significantly higher with a vein anastomosis than with two veins [41]. 

In contrast, several meta-analyses showed a significant correlation with fewer surgical revision procedures when two veins were anastomosed. The additional vein would represent a back-up plan, in case of a venous thrombosis of one vein [42]. The incidence of flap failure was reduced in one study by 36% (p=0.047) [10]. In another study, the flap failure rate was 3.1% in one vein and 1.3% in two veins. The thrombosis rate was 3.1% with a venous anastomosis and 2.3% when two veins were connected. 7.7% of the anastomoses with one vein and 6% of the anastomoses with two veins had to be revised. Therefore, the authors recommend whenever possible to use two venous anastomoses with free flaps [43]. 

Existing connections between the superficial and deep venous system also play an important role. Connections between two deep veins via the superficial system could help to spread the thrombus and occlude both venous anastomosis [44]. In our study, two venous anastomoses were performed whenever possible, due to the easiness and swiftness of coupler-assisted anastomosis. A recent meta-analysis showed reduced venous thrombosis with the use of venous couplers [45]. The eversion of the intima with venous coupling reduces the contact of injured intima with the blood flow and might be one explanation for the reduced thrombosis rates.

## Conclusion

Our study showed a comprehensive analysis of the first 182 free flap reconstructions for extremity reconstruction in a newly established department of plastic surgery. The partial and complete flap loss rates, although higher than the literature mean, is still within the rates of some reported studies, especially considering the high-risk profile of many of the patients in the cohort. This higher rate may also be attributed to the unexperienced, newly constituted reconstructive team, which could be improved through advanced training. Except for the surgical expertise, free flap surgery implies established standards to ensure swift anastomosis failure diagnosis and timely revision. Considering the risk factors for free flap surgery, further randomized controlled studies need to be performed, in order to precisely detect the causality of flap failure.

## Notes

### Competing interests

The authors declare that they have no competing interests.

## Figures and Tables

**Table 1:**
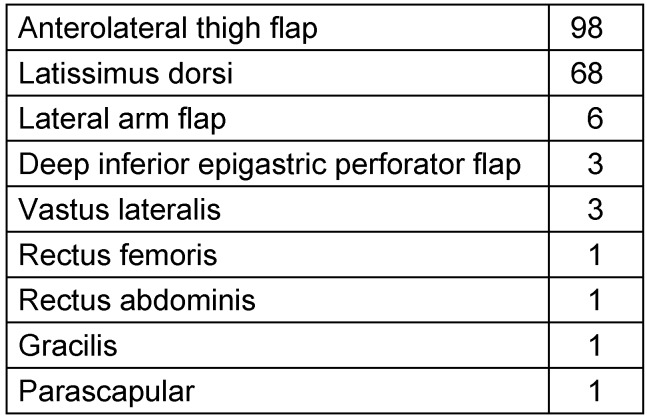
Distribution of performed free flaps

**Table 2:**
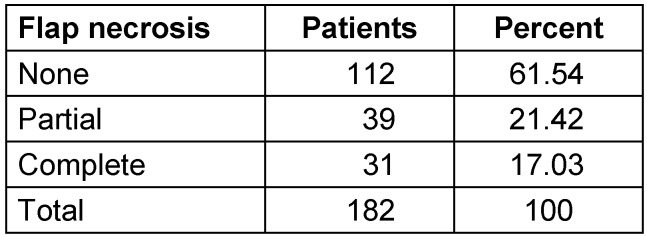
Flap necrosis

**Table 3:**
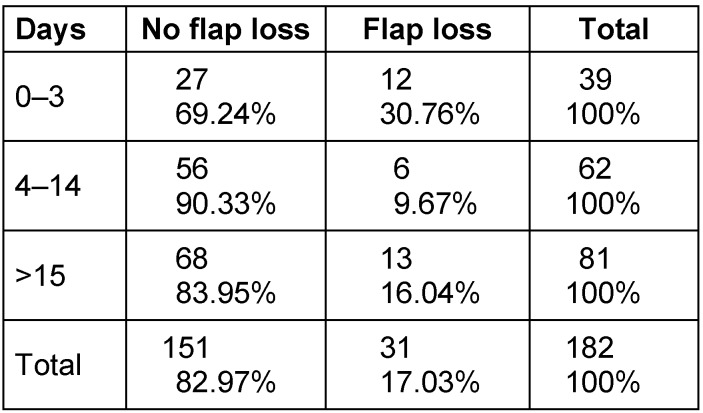
Flap loss according to time elapsed

**Table 4:**
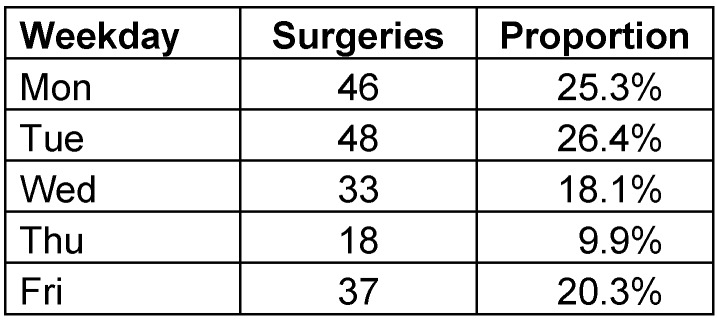
Surgeries according to the day of the week
